# Origin of cancer: what is a (cancer) cell? A systems-complexity perspective - principles of cellular systems [part 1]

**DOI:** 10.3389/fcell.2026.1936518

**Published:** 2026-10-21

**Authors:** Rainer G. Hanselmann

**Affiliations:** IB-Cancer Research Foundation, Saarbrücken, Germany

**Keywords:** carcinogenesis, complex systems, contingency, emergence, EMMI model, integrative pluralism, robustness, self-organization

## Abstract

In tumor tissue, the same mutations are found as in the surrounding healthy tissue; several pediatric malignancies show few or no mutations; and malignant cells can return to an ordered, non-malignant phenotype when their three-dimensional context is restored. Observations of this kind are difficult to reconcile with the reductionist paradigm that, to this day, regards genetic mutations as the necessary and sufficient condition for the malignant transformation of cells. This paper argues that such an approach does not do justice to the biological reality of the cell as a complex system. As a framework for a systemic alternative, the EMMI variables - Energy (E), Matter/Milieu (M), Mechanics/Structure (M), and Information (I) - are proposed as overarching influencing variables of complex cellular systems and, as the central original contribution of this work, as categorizing hubs that assign the nodes of the cellular network to four classes of physical, chemical or biological influences. Based on the concept of integrative pluralism according to Sandra Mitchell as well as the principles of complexity science, key properties of biological systems - such as emergence, dynamics, nonlinearity, variability, robustness, contingency, and convergence - are presented in terms of their significance for cell biology and carcinogenesis. Furthermore, it is discussed why these are more suitable for explaining the development of cancer in the context of complex cells and cellular systems than a purely mutation-centered approach. The present work is primarily aimed at scientists in the life sciences, such as biologists and physicians, and places particular emphasis on the practical relevance of a systemic approach to the initiation and progression of chronic diseases, particularly malignant tumors. A final chapter is dedicated to integrative pluralism as the epistemological foundation for research into complex systems in biomedicine. The framework thereby becomes operationalizable: the choice of an emergent property defines the explanatory goal, and the selection of the external parameters that stand for the EMMI variables determines what becomes visible and assessable. With a good choice, this selection makes it possible to identify those mechanisms that actually determine the development of disease and cancer, and not merely those that accompany it.

## 1 Introduction: why a new perspective on cancer is necessary

“Every object that biology studies is a system of systems.” - François Jacob

Cancer research has made extraordinary progress in identifying molecular mechanisms over the past few decades. The discovery of oncogenes, tumor suppressor genes, and the associated signaling pathways has fundamentally shaped our understanding of carcinogenesis. The somatic mutation theory, according to which accumulated genetic changes represent the necessary and sufficient cause of carcinogenesis, has been considered the central dogma of cancer biology for about 50 years (Weinberg, 2014; Hanahan and Weinberg, 2011). Although this approach has led to significant diagnostic and therapeutic developments, fundamental questions remain unanswered: Why are the same mutations found in tumor tissue as in the surrounding healthy tissue (Huang et al., 2025)? Why are thousands of mutations found in healthy tissue, including in tumor genes (Martincorena et al., 2015)? Why do pediatric malignant tumors show very few or no mutations (Versteeg, 2014; Mack et al., 2014)? Why can tumor cells revert under certain environmental conditions (Short, 2009; Kenny and Bissell, 2003; Weaver et al., 1997)? In this context, I recommend the aforementioned work by Huang et al. (2025), who have dealt with these questions in depth.

These findings point to a fundamental inadequacy of purely reductionist explanatory models. They emphasize the necessity to conceive of the cell in the future as a complex dynamic system - a system whose behavior is determined by the emergent properties of the system as a whole and not by the additive summation of its individual components. Ludwig von Bertalanffy already recognized that biological systems cannot be described by simple linear cause-and-effect chains, but rather constitute hierarchically nested, feedback-controlled systems that actively interact with their environment (von Bertalanffy, 1950; 1968).

Sonnenschein and Soto (2000) summed up this problem succinctly: The Somatic Mutation Theory (SMT) is structurally similar to the geocentric worldview of Ptolemy - a model that was not replaced by a conceptually superior paradigm, but rather artificially kept alive through ever-increasing complexity in the form of epicycles (Huang et al., 2025). Similarly, within the SMT, the list of postulated driver mutations, exception mechanisms, and special molecular cases grows continuously without a coherent mechanistic overall picture emerging. Instead of acknowledging the conceptual limits of the model, it is expanded through increasing differentiation of particular explanations - a symptom of paradigmatic exhaustion in Kuhn’s sense (Kuhn, 1962). By contrast, a systemic understanding of carcinogenesis, which - among other things - emphasizes contingency, emergence, context-dependence, and the dynamic interaction between the cell and its environment under the influence of the EMMI variables, offers not merely a supplement, but a conceptual reframing of the problem.

The philosopher of science Sandra D. Mitchell has developed a conceptual framework in her book Biological Complexity and Integrative Pluralism (2003) as well as in subsequent publications (2008, 2009, 2023) which, in my view, meets these challenges. Her approach of integrative pluralism postulates that complex biological systems cannot be fully captured by a single method or level of explanation, but that complementary perspectives at different levels of organization and abstraction are necessary (Mitchell, 2003). This approach provides the epistemological foundation for the arguments developed in this paper.

In this paper, I lay out the conceptual foundations necessary for a systemic understanding of carcinogenesis. I begin with a definition of the cell as a complex system, introduce the EMMI variables as overarching influencing variables, and discuss in detail the properties typical of complex biological systems: emergence, dynamics and nonlinearity, variability, robustness, contingency, and convergence.

Three of the arguments developed here go beyond a review of complexity-science concepts, and I would like to present them at the very outset so that they are not lost in the broader discussion. First, in Section 11. and in Box 1, I propose to conceive of the four EMMI variables not as hubs in the topological sense but as categorizing hubs: a labelling of the node set of the cellular network that assigns every node to one or several classes on the basis of physical, chemical, or biological influences. This does not turn the EMMI model into a topological claim about degree distributions; it turns a descriptive categorization scheme into an operational one, from which a testable prediction follows (Box 1). Second, in Section 3.5, I argue that the assignment of a molecule or structure to an EMMI variable is context-dependent and that the variables are deliberately conceived as non-orthogonal. This is not an unresolved shortcoming of the model but a requirement that follows from Mitchell’s concept of partiality (Section 12); a scheme with cleanly separated categories would fail to capture the very object it describes. Third, in Section 14, I propose to reframe the somatic mutation theory not as a refuted theory but as a pragmatic law in Mitchell’s sense - a contingent, context-limited generalization that holds in many but not in all cases. Read in this way, the growing list of non-mutational routes to malignancy is no longer a collection of exceptions but the expected consequence of the theory’s scope.

The EMMI variables themselves were introduced earlier (Hanselmann and Welter, 2016; 2022); what is new here is their systematization as categorizing hubs over the cellular network (Box 1), the explicit context dependency of their assignment, and their epistemological grounding in integrative pluralism. Taken together, these three steps turn EMMI from a descriptive vocabulary into an operational systematics. By defining a suitable emergent property together with its associated EMMI parameters/variables, experiments can be designed and analyzed that elucidate, step by step, the initiation and progression of cancer - and, on the same systematic principle, of other diseases as well.

## 2 The cell as a complex system: a systemic definition

### 2.1 Basic characteristics of the cell system

A cell represents the smallest unit of life. It is a structurally distinct, independent, self-sustaining, and self-organizing, nonlinear, heterogeneous whole of functional structures (systems) and individual elements, which forms a complex system that is far from thermodynamic equilibrium. The continuous uptake and release of substances and energy establishes a metabolic process characterized by the synthesis and breakdown of molecules as well as the production, use, and release of energy (Hanselmann and Welter, 2016; 2022; Davies et al., 2013; Prigogine, 1997).

These biophysical properties enable the cell to exhibit the characteristic features of life: (1) structure resulting from high morphological order, (2) responsiveness to internal and external stimuli through adaptation and differentiation, (3) high degree of dynamism of all processes, (4) directed movement and molecular transport, and (5) reproductive capability through proliferation and autopoiesis. Externally, the cell appears as a plastic phenotype that dynamically adapts to its immediate environment.

Ludwig von Bertalanffy defined systems as interactive relationships that are distinct from their environment and, in turn, consist of further interactive relationships (von Bertalanffy, 1950). On this basis, biological systems can be understood as self-organizing functional units that consist of components with different properties and act as a collective whole based on ordered mutual relationships. This system definition is central to understanding carcinogenesis: It implies that changes at the level of individual components - for example, gene mutations - influence but do not determine system behavior. System behavior is an emergent phenomenon that results from the interplay of all components. More on this later.

## 3 The EMMI variables: overarching influencing variables of complex cellular systems

To describe the multiple intrinsic and extrinsic influences acting on complex cell systems and their subsystems in a manageable way, without resorting to a reductionist explanatory framework, Cornelius Welter and I have proposed the EMMI variables. These variables summarize the countless cellular components, processes, structures, and interactions into four overarching influencing variables with which the nodes of the cellular network can be categorized - categorizing hubs in the sense developed in Box 1: Energy (E), Matter/Milieu (M), Mechanics/Structure (M), and Information (I). They do not represent isolated categories, but rather describe access points to a system whose actual functional basis - the Entirety of all Molecular Interactions (EoMI) - cannot, in principle, be fully captured in its complexity (for more details, see Section 12, Integrative Pluralism). Building on this earlier version (Hanselmann and Welter, 2016; 2022), the present work develops the model in two respects. First, I propose to understand the four variables as categories over the nodes of the cell’s scale-free network, and not as hubs within this network. Second, I argue in Section 3.5 that their assignment is necessarily context-dependent and non-orthogonal. When looking at the Somatic Mutation Theory and the other currently discussed hypotheses on the origin of cancer, the EMMI variables are already being applied in isolation here. Protagonists of the SMT see mutations exclusively as the cause (Weinberg, 2014; Hanahan and Weinberg, 2011), others consider the Warburg effect to be causal (Seyfried et al., 2025; Warburg, 1956a; 1956b), and still others see the milieu or the mechanical interaction with the ECM as causal (Soto and Sonnenschein, 2011; Butcher et al., 2009; Levental et al., 2009; Paszek et al., 2005; Soto and Sonnenschein, 2004; Bissell and Radisky, 2001; Wang et al., 2002; Weaver et al., 1997). Due to the reductionist view and the application of only one EMMI variable, none of these hypotheses does justice to a complex cellular system.

Crucially, none of these four variables alone or in direct causality determines cell behavior. They always act in concert, in the sense of nonlinear, reciprocal dependencies, and any change in one variable inevitably entails shifts in the others. Thus, the EMMI model enables a systemic view of cellular functions and their pathological derailment without reducing the genuine complexity of the living to individual molecules or reaction cascades. The advantage of this approach is that it can fundamentally be applied at any system level to gain a conceptual understanding of the system in order to observe and evaluate biological function.

### 3.1 Energy (E)

All cellular processes are energy dependent. The production of energy in the cell (e.g., glycolysis, oxidative phosphorylation) significantly determines the performance and adaptability of the entire cellular system. The amount of ATP provided by the mitochondria influences which molecular processes can be active, which equilibria are established, and which structures are maintained. Cellular work does not arise from the presence of molecules alone, but from the continuous flow of energy through the open system of the cell, which enables these interactions.

In the context of carcinogenesis, the so-called Warburg effect - the preferential use of aerobic glycolysis even when sufficient oxygen is available - is a paradigmatic example of a systemic energy shift that influences all other EMMI variables (Li et al., 2025; Vander Heiden et al., 2009; Warburg, 1956a). The resulting shift in ATP production and localization not only changes the energy status of the cell but also acts directly on the intracellular milieu, on metabolism, on protein homeostasis, and on mechanical properties of the cytoskeleton, which Otto Warburg described in his lecture in Lindau as an increase in disorder (Warburg, 1966). This illustrates that energy is not an isolated control variable, but rather, as a central driver, conditions the entire system of cellular work and must be viewed as such.

Particularly noteworthy in this context is a comparatively new finding regarding ATP: While it was previously assumed that ATP functions almost exclusively as an energy carrier, it has been shown that at physiological intracellular concentrations - in the millimolar range - it acts as a biological aggregation inhibitor for proteins, thereby ensuring the solubility and functionality of a multitude of proteins (Mehringer et al., 2021; Patel et al., 2017). ATP is therefore to be assigned to both the EMMI variable Energy and the EMMI variable Matter/Milieu - an example of the context-dependent assignment of the EMMI variables, which will be discussed in more detail below.

### 3.2 Matter/milieu (M)

The intra- and extracellular milieu - the totality of electrolytes, metabolites, DNA, RNA, proteins, oxygen availability, and pH conditions, to name but a few - is one of the decisive variables for differentiation, function, and phenotype. The extracellular microenvironment constantly interacts with the intracellular milieu via the cell membrane, and thus conveys contextual information that directly and continuously influences molecular activity between the interior and exterior of the cell.

Perturbations of the microenvironment in both areas, such as those caused by chronic hypoxia, acidosis, mutations, or altered nutrient supply, induce system changes that go far beyond direct chemical effects (Hanselmann and Welter, 2016; 2022). Hypoxia, for example, not only reduces oxidative metabolism but also alters the solubility and the tendency of proteins to aggregate due to ATP loss, changes metabolism, influences epigenetic modifications, promotes angiogenic signals, and can contribute to the development of oncogenic phenotypes - without necessarily requiring a gene mutation (Li et al., 2025; Seyfried et al., 2025; Hanahan, 2022; Mehringer et al., 2021).

The relevance of the milieu for carcinogenesis is evident in chronic inflammation as an established risk factor for cancer: The microenvironment characterized by inflammation permanently alters the molecular environment of tissues and can thus contribute to the dedifferentiation of cells and the initiation of malignant transformations (Coussens and Werb, 2002; Balkwill and Mantovani, 2001). Conversely, it has been shown that even an aggressive breast cancer phenotype can be reverted to an ordered, nonmalignant state by restoring a normal three-dimensional milieu (Bissell and Radisky, 2001; Weaver et al., 1997), a finding of fundamental conceptual importance that significantly relativizes the causal role of gene mutations for tumor development.

### 3.3 Mechanics/structure (M)

Morphology, cytoskeleton, organelles, membrane composition, and extracellular matrix (ECM) are not passive carriers of cellular functions, but active control variables. The mechanical properties of the ECM and cell-cell or cell-matrix binding sites significantly determine polarization, metabolism, and gene expression. Tumor cells show characteristic changes in their mechanical properties - increased deformability, altered adhesion properties, and cytoskeletal disruptions - which must be considered not as a consequence, but also as a cause of malignant transformation (Butcher et al., 2009; Lu et al., 2012). Theodor Boveri had already pointed out this influence in 1914 (Boveri, 1914).

The mechanical anchoring of a cell in its environment is essential for its physiological function. The further a cell moves away from its natural “attachment pattern,” the more its ability to perform its physiological tasks and maintain a regular metabolism is compromised. Huang and Ingber described the mechanical properties of the ECM as a possible trigger for malignant dysregulation and urged that mechanics be taken seriously as an independent control variable of cell biology:

*“brings solid-state mechanotransduction on a par with oncogenic signaling pathways in malignant transformation”* (Huang and Ingber, 2005)

Particularly impressive in this context is the finding that tumor cells cultured on soft substrates or suitable gel layers begin to differentiate and return to a more normal proliferation behavior (Kenny and Bissell, 2003; Weaver et al., 1997). This reversibility of a malignant phenotype solely through changes in mechanical conditions or the cellular context - without correcting a single gene mutation - underscores the systemic importance of the Mechanics/Structure variable.

In addition, recent work shows that the hydraulic instability of a cell can directly influence its decision to undergo apoptosis (Chartier et al., 2021), and that mechanical forces modulate enzyme activities (Bao, 2009). Cell mechanics is thus far more than a biophysical boundary condition - it is an independent, causal influencing variable on differentiation and cell behavior.

### 3.4 Information (I)

In the cell biological context, information encompasses all information-bearing molecules: DNA, RNA, proteins, lipids, and metabolites in their function as carriers of intrinsic information. Every molecule carries its own information, and any change in its composition, conformation, or energy charge alters its information content and thus its function in the network of the cell system (Hanselmann and Welter, 2016; 2022). Gene mutations are therefore changes in genetic information, but they represent only a subset of all informational influencing variables that govern the cell system (Karsenti, 2008; Frieden and Gatenby, 2011).

This becomes particularly clear when considering intrinsically disordered proteins (IDPs): These proteins - which are widespread in the human proteome and overrepresented in tumor cells - do not possess a stable three-dimensional structure, but rather move within a broad “conformational space.” Their function is not determined solely by the coding genes, but is strongly dependent on environmental conditions such as ATP concentration, pH value, temperature, and the composition of the molecular environment (Uversky, 2017; Alberti et al., 2019). The classical concept of a fixed sequence-structure-function relationship is fundamentally challenged here.

Furthermore, findings from epigenetics have significantly relativized the monocausal role of the DNA sequence as the sole source of information for the cell: histone modifications, DNA methylation, and regulation by non-coding RNAs make it possible to inherit phenotypes across cell divisions without the DNA sequence being changed (Waddington, 1942; Ashe et al., 2021). In fact, during somatic cell division, not only is the DNA replicated and equally distributed between the two daughter cells, but all other cell components are also duplicated in the current context in which the cell finds itself. After cell division, the two daughter cells thus inherit not only an identical set of chromosomes but the identical intracellular equipment as their mother cell, which allows them to interact directly with the existing extracellular space and microenvironment (context) just as their mother cell did. Inheritance as an important component of evolution is therefore not only carried by the genome, but to a certain extent also by the existing molecules and their established dynamic interactions in the cell system, as well as the stable cell environment, its context. In today’s cell culture methods, this fact is used as a matter of course. To maintain the phenotype of a cell, cells are expanded in specially coated cell culture flasks or cultivated directly in special 3D cell culture matrices. Interesting in this context is that in hematopoietic stem cells, the endo-lysosomal vesicles - lysosomes, autophagosomes, recycling endosomes, and the cytoplasm volume - are specifically distributed asymmetrically: the daughter cell with more lysosomes remains in the stem cell state, while the daughter cell with fewer lysosomes, more cytoplasm, and more active mitochondria proceeds to differentiation. The cytoplasmic equipment thus directly determines how daughter cells are metabolically activated and in what way they interact with their environment (Bera and Loeffler, 2025; Loeffler et al., 2019).

### 3.5 Context dependency and conceptual flexibility of the EMMI assignment

It is important to emphasize that the assignments in the EMMI model are context-dependent and flexible. ATP, for example, primarily belongs to the variable Energy, but also functions as a significant solubilizer for proteins and, in this context, is to be assigned to the variable Matter/Milieu. p53, which is classically considered a tumor suppressor gene (Information) and assigned to cell repair, simultaneously regulates the energy metabolism of mitochondria, autophagy, and redox homeostasis - and thus touches upon all four EMMI variables (Kruiswijk et al., 2015). The ECM as a mechanical environment (Mechanics/Structure) accumulates growth factors and signaling molecules (Matter/Milieu), which are released when needed (Naba, 2024; Bonnans et al., 2014; Taipale and Keski-Oja, 1997). The basement membrane appears to adsorb large amounts of protons due to its high electronegativity (Donnan effect) and thus intervenes in the regulation of the pH value (Durbin and Renden, 2024; Aydogan Gokturk et al., 2022; Mattern et al., 2008).

This apparent ambiguity is not a conceptual flaw of the model, but reflects the genuine contingency of biological systems. The EMMI variables are not sharply delimited domains, but descriptive frameworks that allow the essential systemic influences on cellular work to be named and weighted, and disruptions to be localized. Precisely the non-orthogonality of the variables makes it clear that every pathological change in the cell system - whether energy deficiency, milieu change, mechanical dysregulation, or information disruption - propagates through the entire system architecture of the cell and cannot be reduced to a single causal factor.

## 4 Self-organization: the fundamental principle of cellular complexity

A complex cell system is characterized by the fact that it spontaneously structures and organizes itself. Control of the self-organizing system is not localized in a central authority, but is distributed among the elements of the system and defined by their interactions and feedback loops. In the case of a cell, these are all the molecules and chemical elements that make up a cell (the Entirety of all Molecular Interactions, EoMI).

A paradigmatic experiment for visualizing cellular self-organization was conducted by Cheng and Ferrell (2019): *Xenopus* oocytes were homogenized, and the homogenate was observed under a microscope. Spontaneously, through energy consumption, the original cellular structures - cell shape, endoplasmic reticulum, spindle apparatus - reconstituted themselves. This experiment impressively demonstrates that the information for structure formation does not need to be provided externally, but is intrinsically encoded in the system. Self-organization is therefore not an emergent exception, but the basal principle of cellular existence.

Using models in his seminal work The Origins of Order (1993), Kauffman showed that self-organization in complex networks is a universal property that arises independently of natural selection. Boolean networks that exceed a certain connectivity threshold spontaneously organize themselves into ordered attractors (Kauffman, 1993; Kauffman, 1995). This insight is of fundamental importance for cancer research: If self-organization is an intrinsic property of biological networks, then tumor cells cannot arise solely through the presence of certain mutations - rather, there must be systemic changes that shift the attractor of the cell system.

Mitchell (2023) describes self-organization as one of the central mechanisms through which complex emergent properties arise at higher system levels, a process that cannot be explained simply by summing the properties of the individual components. In cancer biology, this manifests itself in the hallmark of tumor tissue, which - despite massive molecular heterogeneity - develops stable collective properties: invasive growth, angiogenesis, immune evasion (Hanahan and Weinberg, 2011).

## 5 Emergence: when the whole is more than the sum of its parts

### 5.1 Definition and biological relevance

The term emergence refers to the appearance of system properties that cannot be predicted on the basis of the properties of the individual components and their known interactions (cf. Aristotle, 384–322 BCE). Mitchell defines three core characteristics of emergent phenomena: (1) novelty of properties at the macro level, (2) unpredictability from the properties of the micro level, and (3) - in its strongest form - the capacity for downward causation, where the emergent macro property in turn influences the behavior of the micro components (Mitchell, 2023; McLaughlin, 1992).

It is important to distinguish between weak and strong emergence. In weak emergence, higher-level properties can in principle be explained in terms of micro-level properties, but require knowledge of the interactions between the components. In strong emergence, genuine downward causation is additionally at play, which cannot be predicted or derived from the properties of the micro-level components (Bedau and Humphreys, 2008; Mitchell, 2012).

Both forms of emergence can be observed in cell biology. A cell’s ability to migrate is an example of weak emergence: It results from the coordinated activity of the cytoskeleton, membrane receptors, signaling cascades, and energy metabolism, but can in principle - though not entirely in practice - be derived from these components. P. C. W. Davies defines strong emergence as the ability of all somatic cells of a eukaryotic organism - despite having identical DNA - to differentiate into many different cell types - such as liver, kidney, brain, skin - during development as a response to a network of physical and chemical environmental signals (Davies, 2012). The emergence of an invasive tumor phenotype from an ensemble of genetically heterogeneous cells also shows characteristics of strong emergence: The collective invasion phenotype influences the behavior of individual tumor cells through remodeling of the extracellular matrix and secretion of paracrine factors - a genuine downward causation, as strong emergence should show (Friedl and Alexander, 2011; Couzin and Krause, 2003).

### 5.2 Emergence in cancer biology: collective behavior and tumor heterogeneity

Tumor tissue is a paradigmatic example of emergent biological complexity. Groups of cancer cells exhibit behavior that is fundamentally different from that of individual tumor cells. Tumor cells in a cluster exhibit properties such as collective migration, resistance to chemotherapeutic agents, angiogenesis induction, and immune evasion, which none of the individual cells involved exhibit on their own (Friedl and Alexander, 2011; Marusyk et al., 2012; Tabassum and Polyak, 2015; Gao et al., 2025). According to Alladin and colleagues, tumor progression even seems to depend on whether or not a single transformed cell is surrounded by other transformed cells (Alladin et al., 2020).

Intratumoral heterogeneity - one of the greatest challenges of modern oncology - can be understood from a systemic perspective as an expression of emergent complexity. Genetically and phenotypically different cell populations within a tumor interact via paracrine signals, metabolic products, mechanical forces, and a constantly adapting microenvironment, thus creating a dynamic ecological system whose overall behavior cannot be derived from the properties of the individual clones (Marusyk et al., 2012; Tabassum and Polyak, 2015).

Mitchell (2023) points out that self-organization dynamics with positive and negative feedback loops generate and stabilize emergent properties at higher levels of the system - properties that are not possessed by any of the individual components and do not represent the simple sum of the properties of those parts. This understanding of emergence is of immediate practical relevance for cancer therapy: if the tumor phenotype is an emergent property of the overall system, therapies that target only individual molecular targets will be systemically insufficient - which explains the clinical experience of unsatisfactory tumor therapies.

## 6 Dynamics and nonlinearity: cellular systems in flux

### 6.1 Dynamic complexity in living systems

Complex systems such as cells are dynamic systems that continuously interact with their environment. They take in matter and energy precursors from their immediate microenvironment to maintain structure and order - a state that Prigogine described in a model as a dissipative structure: a highly organized structure that is stabilized by a permanent flow of energy far from thermodynamic equilibrium (Prigogine, 1997).

Mitchell describes dynamic complexity as a trajectory of state changes represented by nonlinear, chaotic functions (Mitchell, 2023). In cell biology, this manifests in the heterogeneous dynamics of cellular processes: feedback loops of different time constants - from milliseconds in ion channels to hours and days in gene expression changes - superimpose to form a high-dimensional dynamic system that is not readily amenable to linear analyses (Tyson et al., 2003; Novak and Tyson, 2008).

Particularly important is the realization that the proportion of chaotic ordering processes increases with the increasing complexity of a system. This means that minor modifications in a highly complex system - for example, a single gene mutation in a scale-free signaling network - can lead to significant, seemingly unpredictable changes in state. This behavior could potentially explain the sometimes dramatic effects seen in hereditary diseases that are based on a single mutation. This nonlinearity is not a malfunction of the system, but is essential for its plasticity and adaptability.

### 6.2 Nonlinear equilibria and cellular state transitions

Biological systems are characteristically in nonlinear dynamic equilibria. The concept of attractors from the theory of dynamic systems offers a powerful descriptive tool: cell types can be understood as attractors in a high-dimensional state space (the “Waddington epigenetic landscape model”). Normal differentiation processes correspond to the transition between stable attractors; malignant transformation corresponds to entering a new, pathological (unstable) attractor (Huang et al., 2009; Kauffman, 1993; Waddington, 1957).

The systems physicist Prigogine has shown that far from thermodynamic equilibrium, systems can abruptly transition into new states due to fluctuations or external perturbations (phase transitions) - a phenomenon referred to in biological systems as bifurcation (Prigogine, 1997). In cancer biology, such bifurcations correspond to events where a multifactorially stressed cell system tips into a malignant attractor - not necessarily triggered by a single mutation, but by the cumulative destabilization of the system equilibrium, for example, through chronic inflammatory processes (Hanselmann and Welter, 2016).

In an influential paper, Tyson, Chen, and Novak systematically described the nonlinear dynamic properties of cellular regulatory networks and showed how bistability, oscillations, and hysteresis represent fundamental mechanisms of cellular decision-making processes (Tyson et al., 2003). These findings are of direct relevance to cancer research: the nonlinearity of cellular networks explains why the transition to malignancy often occurs abruptly, and why targeted individual interventions in such networks often show only limited therapeutic efficacy.

## 7 Variability: heterogeneity as a systemic necessity

### 7.1 Biological variability at the cellular level

Variability is an indispensable feature of all biological systems. Mitchell emphasizes that it is a necessary component of evolutionary dynamics and exists at all levels of biological organization - from the molecular to the cellular to the population level (Mitchell, 2008).

At the cellular level, variability manifests in the phenotypic heterogeneity of genetically identical cells. In a fundamental experiment, Elowitz and colleagues demonstrated in E-coli that stochastic fluctuations in gene expression lead to significant variability in the behavior of genetically identical individual cells (Eldar and Elowitz, 2010; Elowitz et al., 2002). This stochastic gene expression is not a system malfunction, but a functional feature that is regulated and utilized by feedback loops. It enables the cell population to react to unexpected environmental changes without every single cell having to carry all possible responses within itself in a preformed state.

In cancer research, intratumoral variability - the phenotypic and genetic heterogeneity within a single tumor - is one of the most difficult obstacles to overcome for successful therapeutic concepts (Marusyk et al., 2012; Greaves and Maley, 2012).

### 7.2 Variability as an evolutionary resource in tumor progression

From a systemic perspective, intratumoral heterogeneity is an emergent property of the tumor system that arises from the same mechanisms that also generate normal biological variability: stochastic gene expression, epigenetic diversification, and nonlinear responses of the Entirety of all Molecular Interactions (EoMI) to environmental signals. This variability creates the raw material from which therapy-resistant clones can emerge through intratumoral selection (Hanselmann and Welter, 2022; Marusyk et al., 2020; Greaves and Maley, 2012).

In her analysis of biological complexity, Mitchell describes that variability and historical contingency are fundamental properties of complex evolutionary systems that cannot be described by universal, exceptionless laws (Mitchell, 2000; Mitchell, 2003; Mitchell, 2023). For cancer therapy, this means: A therapy based on the assumption of a uniform tumor phenotype is systemically inadequate. Modern personalized therapy approaches - which are only marginally based on individual tumor microenvironments, mutation profiles, and immune landscapes - are an expression of the practical consequence of a systemic understanding of variability, but are insufficient because they are based only on the use of information and do not take other essential therapeutic influencing variables such as energy, environment, and mechanics into account.

## 8 Robustness: stability and adaptability of complex systems

### 8.1 Biological robustness as a systemic property

Biological robustness is the property of a living system to maintain its function and structure in the face of internal and external perturbations. Kitano identified robustness as a fundamental feature of complex biological systems and showed that it is achieved through redundancy, modularity, and diverse feedback loops (Kitano, 2004a); his interpretation of the tumor as a robust system in itself is also relevant to the understanding of malignant processes (Kitano, 2004b).

In this context, I would like to point out a possible extension of these features that results from the structural and functional diversity of molecules. As early as 1999, Jeffery described so-called moonlighting proteins, the existence of which has since been confirmed and systematically cataloged in several papers (Werelusz et al., 2024; Jeffery, 2003; Jeffery, 2018; Mani et al., 2015; www.moonlightingproteins.org). Using the example of glyceraldehyde-3-phosphate dehydrogenase (GAPDH), Sirover shows how a single polypeptide assumes functionally distinct tasks depending on its subcellular localization: membrane-bound GAPDH is involved in membrane fusion, endocytosis, and iron transport; cytoplasmic GAPDH is involved in glycolysis, regulates mRNA stability, and is involved in ER-Golgi transport; nuclear GAPDH is involved in apoptosis, transcriptional gene regulation, the maintenance of DNA integrity, and nuclear tRNA export (Sirover, 1999; 2011; 2018). The resulting increase in functional variability illustrates the diversity with which cells can robustly react to perturbations via the Entirety of their Molecular Interactions (EoMI) (Hanselmann and Welter, 2022).

This multi-level perspective corresponds to the program of integrative pluralism as advocated by Mitchell: Complex biological phenomena cannot be understood through a single, privileged level of explanation, but require the integration of several complementary models and levels of causation to explain the specific case (Mitchell, 2003; Mitchell, 2023). In doing so, Mitchell explicitly ties in with Kitano’s concept of robustness and argues that robust system dynamics suggest a - non-reductive, compatible with energy conservation - concept of downward causation, in which the organization of the systemic level restricts and contextualizes the behavior of the individual components (Mitchell, 2023). In Mitchell’s terminology, this marks the area where weak emergence transitions into strong emergence. Examples of such multi-level robustness and regulatory dynamics can be found at different biological levels: trauma-induced neuronal reorganization in the brain, the change in gene expression in the honeybee triggered by social conditions (Robinson et al., 2005), as well as the cellular factor situation radically changed by the position in a social hierarchy in cichlids (Fernald and Maruska, 2012); Mitchell cites them as evidence that robustness is to be understood as a distributed, multi-level system property (Mitchell, 2023; cf. generally Stelling et al., 2004).

Against this background, the EMMI framework does not see itself as a unifying super-theory, but as an integrative classification scheme in the sense of Mitchell: a heuristic that bundles the multi-level causes of cellular behavior into four overarching variables - energy, matter/milieu, mechanics/structure, and information - without reducing one of these variables to another (BOX 1). In this framework, robustness is the system’s ability to respond to the perturbation of one or more EMMI variables through adaptive reactions in the remaining variables and to establish a new dynamic equilibrium. This cohesive system behavior explains why normal tissue cells compensate for minor perturbations, while massive or chronic disruptions lead to irreversible state changes.

### 8.2 Robustness and therapy resistance in cancer biology

The robustness of the tumor system against therapeutic interventions is one of the central clinical problems in oncology. From a systemic perspective, therapy resistance is not primarily a genetic phenomenon - although mutations in target structures undoubtedly play a role - but an expression of the systemic robustness of the tumor network. The finding that in about 30% of knockout experiments, the silencing of a target gene does not produce a significant phenotype is paradigmatic: the network reorganizes itself through alternative pathways to maintain the original function (Mitchell, 2023).

For cancer therapy, this implies that strategies aimed at the simultaneous perturbation of multiple hubs in the tumor network should be systemically superior to the blockade of individual molecular targets. The clinical experience of combination chemotherapy, combined targeted therapies, and especially immune checkpoint inhibition - which addresses the tumor-immunological network at a systemic level - supports this systemic view (Topalian et al., 2016; Postow et al., 2015), but does not do it justice under the current approach.

## 9 Contingency: why the history of a cell determines its phenotype

### 9.1 Contingency in biological systems

Contingency describes all the possibilities that a system has at a defined point in time, which, however, do not necessarily have to be pursued. This shows the dependence of the system state on its historical development. Mitchell has positioned this term centrally in her analysis of biological laws: Biological causal relationships are not universally and unconditionally valid - as classical physics demands for fundamental laws - but are dependent on the historical conditions under which a system has emerged (Mitchell, 2000; Mitchell, 2003; Mitchell, 2023).

At the cellular level, contingency manifests itself in the path dependence of cellular systems: The current state of a cell depends not only on its current molecular conditions but also on its developmental history, its division history, and the cumulative effects of past events. This phenomenon is discussed in modern cell biology under the terms cellular memory, epigenetic inheritance, and transgenerational epigenetics (Jablonka and Lamb, 2005; Heard and Martienssen, 2014).

A particularly clear example of such path dependence is developmental polyploidization: hepatocytes, cardiomyocytes, and megakaryocytes acquire additional complete chromosome sets as a programmed step of differentiation, and this alteration in genomic dosage permanently shapes their size, metabolism, proliferative capacity, and stress response (Darmasaputra et al., 2024; Donne et al., 2020; Derks and Bergmann, 2020).

What significance do the EMMI variables have in this context? Contingency points to the fact that the system state depends on its historical development. The EMMI variables are the mechanisms that are significantly influenced by this historical context. This means that their reactions to the most diverse circumstances and events to which a cell is exposed during its life determine the current context in the cell’s present, and it is their reaction to events that determines the present and future context.

When cancer cells develop, we must therefore consider the state of the EMMI variables that existed at the time when the cell was still “normal.” If external cancer-causing noxious agents now act on the tissue, this leads to changes to which the tissue and its cells react. If the noxious agent is, for example, a non-mutagenic chemical substance, chronic exposure could lead to the development of a chronic inflammatory focus. Depending on the extent, the cell would now react to this state with all four variables in order to stabilize itself. The morphological and metabolic variability that cells can assume under such circumstances is considerable. Clinically, this is shown in the various degrees and heterogeneity of dysplasia, which, if the inflammation disappears, can be completely reversible. However, if the inflammation persists, the cells change further and promote the possibility that a malignant cell develops from them.

This highlights a problem that can occur when applying this model: the problem of the arbitrariness of the model. In this regard, I refer to Section 12 (Integrative Pluralism), in which I address the partiality of the model and thus justify its applicability. It becomes clear that generally valid, context-independent EMMI classifications are not possible - and this is not a flaw of the model, but a realistic reflection of the genuine contingency and its influencing variables in biological systems.

### 9.2 Contingency and carcinogenesis: why the same trigger produces different tumors

The contingency dependence of cellular systems provides an explanation for one of the most puzzling findings in clinical oncology: The same genetic trigger - for example, a KRAS mutation - leads to fundamentally different tumor phenotypes in different cell types. The KRAS-mutated pancreatic adenocarcinoma differs fundamentally - molecularly, clinically, and therapeutically - from the KRAS-mutated colorectal carcinoma, although the initial genetic lesion is identical (Ryan et al., 2014). The converse case - an identical, ubiquitously expressed lesion that nevertheless manifests in only a few tissues, as with BRCA1 – see 11.4 (Monteiro, 2003; Elledge and Amon, 2002).

From a systemic perspective, this is explained by the contingency of the system states: The tissue context, the differentiation history of the affected cells, and the cellular microenvironment or cell context determine which pathological attractor the system will tip into after the mutation occurs. In this model, mutations are not necessarily primary causes of carcinogenesis, but perturbations whose systemic consequences depend on the overall configuration of the EMMI system at the time of the mutation (Hanselmann and Welter, 2016; Hanselmann and Welter, 2022).

This aspect also became clear in studies on atherosclerotic tissue. Pan et al. (2024) showed that the conditional expression of the oncogenic mutation KRAS-G12D - one of the most frequent KRAS mutations in malignant tumors - in smooth muscle cells enhances tumor-like properties and genomic instability. This is a contradiction, since this tissue practically never degenerates into malignancy and does not metastasize despite such oncogenic drivers and tumor-like features (Chatterjee and Martin, 2024).

## 10 Convergence: how different paths lead to similar phenotypes

In complex systems, the concept of convergence describes the phenomenon where different initial states or perturbation scenarios can lead to the same or similar end states (attractors) if feedback loops are sufficiently strongly interconnected. In evolutionary biology, functional convergence has long been known: vertebrates and invertebrates have independently developed camera-like eyes (Land and Nilsson, 2012).

In cancer biology, convergence manifests in an important phenomenon described as phenotypic convergence or functional convergence: tumor cells of different genetic origins converge in culture and *in vivo* on similar metabolic, proliferative, and invasive phenotypes when placed in the same microenvironmental context.

In the EMMI framework, convergence is an expression of the cohesive force of the cellular system: all EMMI variables strive to establish a new dynamic equilibrium, and depending on the microenvironment attractor in which they are located, they converge on similar system states - regardless of the specific molecular perturbation pathway. This explains another clinical observation: tumor cells can return to a normal phenotype after transfer from a tumor microenvironment to a physiological environment (Bissell and Hines, 2011; Bissell and Radisky, 2001; Weaver et al., 1997).

For systems biology and cancer therapy, convergence means that therapeutic approaches that normalize the tumor microenvironment (EMMI variable Matter/Milieu) may be more efficient than blocking individual molecular pathways, as they shift the systemic attractor toward which all cellular networks converge. This approach is increasingly receiving clinical attention in modern tumor microenvironment therapy (TME therapy).

## 11 Scale-free networks: the architecture of cellular complexity

### 11.1 Structure and properties of scale-free networks

To formally describe the behavior and properties of cellular systems, the concept of the network is helpful. In an abstract network representation, molecules and structures of a cell are represented as nodes, and the relationships existing between them - regardless of their biochemical nature - as connecting lines (edges). Analyses of cellular networks show that they exhibit scale-free properties: that is, there is no preferred node size; the distribution of connections follows a power law (Barabási and Oltvai, 2004; Barabási and Albert, 1999).

The characteristic features of scale-free networks are: (1) The majority of nodes have few connections; (2) a small number of nodes - the hubs - have a disproportionately high number of connections; (3) scale-free networks are robust against random failure of nodes (since hubs are rarely affected), but vulnerable to targeted attacks on hubs (Barabási and Albert, 1999).

In the cellular context, the most prominent hubs - e.g., molecules like ATP, NADH, or water - are capable of massively influencing the entire system. If ATP were removed from a cell, it would immediately lead to cell death; a perturbation of a node with few connections, however, can be absorbed through systemic compensation (robustness) (Barabási and Oltvai, 2004; Jeong et al., 2001). It should be noted, however, that the empirical claim that biological networks are scale-free does not withstand strict statistical testing in many data sets; log-normal distributions often fit at least equally well (Broido and Clauset, 2019; Lima-Mendez and van Helden, 2009). The argument developed here therefore does not rest on a strict power-law topology, but on the weaker and better-supported observation that cellular networks are strongly heterogeneous in degree.

### 11.2 Why the EMMI variables are not classical hubs but classes over nodes

I propose here to regard the four EMMI variables as categorizing hubs of the cellular network. A categorizing hub is not a node of the network and has no degree of its own; it is a class over nodes that groups together those elements whose function, in a given context, is primarily determined by Energy, Matter/Milieu, Mechanics/Structure, or Information. The actual hubs of the network are not replaced by this level, but rather localized within the categories (the full argument, as well as the two ways in which the categorization can be made usable, are given in BOX 1). Energy (as the category grouping all ATP-dependent processes), Matter/Milieu (as the category grouping the intracellular milieu and all metabolites), Mechanics/Structure (as the category grouping all structural components from the ECM to the cytoskeleton), and Information (as the category grouping all signaling molecules and genetic regulators) are universally involved in all cellular processes.

The EMMI model is thus not merely a descriptive but an operational categorization scheme. A perturbation confined to one EMMI category propagates into the others because the nodes that the categories group together are themselves densely connected in the network - the coupling is a property of the underlying network, not of the categorizing level. This systemic coupling of the EMMI variables explains why pathological processes such as hypoxia profoundly affect the entire network, as they always affect all four dimensions simultaneously: energy metabolism (e.g., ATP depletion), the milieu (e.g., lactate accumulation, pH drop), mechanics (e.g., cytoskeleton remodeling, cell edema), and information (e.g., HIF-1α activation, gene expression changes) (Semenza, 2012).

That the assignment of nodes to these categories is not fixed but can be dynamically reconfigured is shown by polyploid giant cancer cells (PGCCs): under chemotherapy, irradiation, or hypoxia, a subpopulation of tumor cells undergoes genome doubling and, after depolyploidization, generates progeny with increased adaptive fitness (Ogawa et al., 2025; Song et al., 2021). Crucially, this does not concern the variable Information alone: the formation of PGCCs is accompanied by altered metabolic programs, prolonged drug retention and dormancy, as well as a drastically altered cell and nuclear geometry, and thus affects all four EMMI variables simultaneously (Ogawa et al., 2025; Pudełek et al., 2024). Heng and Heng interpret the underlying process as stress-induced generation of new information at the level of the overall system (Heng and Heng, 2022). For the model proposed here, PGCCs therefore represent a test case: it predicts that a perturbation confined to a single EMMI category will be compensated, whereas the simultaneous perturbation of several categories should destabilize this attractor.

*Note: How did hubs evolve?* The emergence and development of hubs in early eukaryotic cells was a process of continuity and leaps. The preferential attachment theory of Barabási and Albert explains why older network nodes tend to become preferred connection partners for new elements: the more connections a node already has, the more attractive it is for new partners (Barabási and Albert, 1999). A particularly impressive example of an evolutionary leap in the establishment of cellular hubs is the endosymbiotic union of the eukaryotic precursor cell with a mitochondrial precursor: as a result, the cell suddenly had access to a drastically increased ATP production (32 instead of 2 ATP molecules per glucose molecule), which fundamentally strengthened the nodes grouped under the EMMI variable Energy and massively promoted the development of all ATP-dependent mechanisms.

### 11.3 The EMMI variables and the concept of scale-free networks - categorizing hubs

The image of the cellular network suggests conceiving of the four EMMI variables as particularly highly connected nodes - as hubs. On closer examination of the foundations of network theory, this reading does not hold. Something else, however, is possible and productive: to assign the nodes of a cellular network to the EMMI variables, that is, to categorize them. For this role, I introduce here the term categorizing hub - a term that network theory does not know.

#### 11.3.1 Why the EMMI variables are not classical hubs in the network-theoretical sense

In network theory, a hub is not a node distinguished by its content, but a measured node: an element of the same graph that differs from all other nodes solely by its degree of connectivity - the number of its edges - and whose emergence is explained by network growth and preferential attachment (Barabási and Oltvai, 2004; Barabási and Albert, 1999). Two objections to an identification with the EMMI variables follow from this.

First, a difference in kind. The EMMI variables are not molecular entities. They have no edges, no degree, and no position in the graph. They are classes over nodes, not nodes. Were they drawn as nodes, the resulting lines would not be physical, chemical, or biological interactions, but membership relations - two categorically different things in one diagram.

Second, a statistical objection. A category node would be, by stipulation, connected to thousands of nodes; it has not arisen through growth, as is usual in typical networks. The degree distribution that defines scale-freeness would thus not be tested but stipulated. The hub status would be a consequence of the drawing and not a finding. Whether cellular networks are scale-free in the strict statistical sense is contested in any case (Section 11.1); the argument developed here does not depend on it.

#### 11.3.2 The concept of the categorizing hub

A categorizing hub is not a node, but an access point at the level of classes. It groups together those nodes of the cellular network whose function, in a given context, is primarily determined by Energy, Matter/Milieu, Mechanics/Structure, or Information. Formally, the EMMI categorization is an assignment applied to a set of nodes, and not an extension of the graph: the topology of the network remains unchanged whether or not the categorizing level is drawn in. The actual hubs are not replaced by it - they lie within the categories.

The closest already-established concept is hierarchical modularity (Ravasz et al., 2002) or the functional module (Hartwell et al., 1999). The difference is essential: modules are defined topologically, through clustering within the graph. EMMI categories are defined by the kind of physical, chemical, or biological influence at work, regardless of whether the corresponding nodes are neighbors in the graph. Precisely for this reason a separate term is needed - network theory has no name for a level of this kind.

The status of the categorization is therefore heuristic and epistemic, not topological. Its value lies in reducing a system that cannot in principle be fully captured - the Entirety of all Molecular Interactions (EoMI) - to four nameable access points, and thereby making it addressable. This is partiality in Mitchell’s sense (2003, 2023): every representation leaves things out, and by leaving them out, it selects. The EMMI categorization makes this selection explicit and thus testable.

### 11.4 Three levels of contingency, their assignment - and when the category itself changes

A three-level systematics of the contingency of molecular nodes is proposed in the following. It is to be understood as a theoretical ordering model with which different forms of biological context-dependence can be distinguished within the EMMI approach developed here. The three levels therefore do not represent an already-established biological classification, but a proposal to order empirically known forms of context-dependence systematically according to whether the strength, the direction, or the functional identity of a node changes.

The necessity of such a context-dependent view is biologically well founded. Across the spectrum of cancer driver genes, tissue specificity is not the exception but a fundamental feature of tumor biology (Haigis, Cichowski, and Elledge, 2019). Sack et al. (2018) were moreover able to show that even fundamental programs of proliferation control are pronouncedly cell- and tissue-specifically wired. It does not yet follow from this, however, which type of contingency underlies an observed tissue specificity. It is precisely this distinction that the following systematics is intended to enable.

A molecular node can be represented, in simplified form, by three specifications: which categories it belongs to, with what weight it enters each of these categories, and with what sign its respective state acts on the malignant phenotype. Each of the three proposed contingency levels describe one of these quantities.

#### 11.4.1 Level 1 - weight contingency

In weight contingency, a node remains assigned to the same functional categories, and the basic direction of its effect is also preserved. What changes, in a context-dependent manner, is how strongly the individual anchorings weigh for the resulting phenotype. The multiple assignment of a node - its non-orthogonality - thus persists; what is variable is its relative functional significance.

BRCA1 may serve as an example, whereby its classification here must expressly be understood as an interpretation within the proposed model and not as an experimentally established EMMI classification. BRCA1 is primarily an information node, in particular on account of its central functions in homologous recombination, DNA repair, and genome stability. At the same time, functional relationships to further system levels exist. Thus BRCA1 influences the energy metabolism of breast cancer cells; its restoration can bring about a metabolic reprogramming with reduced glycolysis and enhanced oxidative energy production (Privat et al., 2014). Furthermore, Sasanuma et al. (2018) show that BRCA1 has a special importance in the removal of estrogen-induced pathological topoisomerase II-DNA complexes, thereby establishing a mechanistic connection between hormonal load, replication stress, and genome stability.

These findings are compatible with the hypothesis that the functional significance of a BRCA1 defect in breast and ovarian tissue is weighted more strongly than in numerous other tissues on account of additional hormonal, proliferative, and metabolic loads. The pronounced tissue specificity of tumor predisposition given a simultaneously ubiquitously required BRCA1 function has long been known and has repeatedly been highlighted as a biological phenomenon in need of explanation (Elledge and Amon, 2002; Monteiro, 2003).

What would not be sufficiently substantiated, by contrast, is the stronger claim that all of these additional couplings have already been causally demonstrated as the explanation of the breast and ovarian specificity. In particular, findings on matrix stiffness and mechanically induced DNA damage in the mammary epithelium (Jones et al., 2025) document important relationships between mechanics, metabolism, and genome stability, but do not demonstrate a specific mechanical BRCA1 coupling. In the sense of the proposed model, BRCA1 can therefore be interpreted as an example of the fact that a fundamentally similar molecular defect can contribute to different degrees to the malignant phenotype depending on the tissue context.

This level answers the question:

*How strongly does the same node contribute to the phenotype in a given context?*

Experimentally, weight contingency would be assumed when, for the same molecular state, different effect strengths at a constant direction of effect can be demonstrated in different contexts.

#### 11.4.2 Level 2 - sign contingency

The second proposed level goes beyond a change in effect strength. In sign contingency, the node remains assigned to the same functional category, but the direction of its contribution to the malignant phenotype changes. By “sign” is meant here whether a particular state or a particular activity of the node promotes the emergence or progression of the malignant phenotype (+) or counteracts it (−).

A particularly suitable example of this is the NOTCH signaling pathway. NOTCH remains, independently of the respective tissue, a regulatory signal-transduction or information node. Its effect on tumor development and tumor progression can, however, be fundamentally different depending on the cellular and tissue context. In T-cell precursor cells, an aberrant activation of NOTCH1 can act in a pronouncedly oncogenic manner and is a central pathogenetic mechanism of T-cell acute lymphoblastic leukemia (T-ALL). In various squamous epithelia, by contrast, the NOTCH signaling pathway can take on tumor-suppressive functions, among other things by promoting differentiation and counteracting uncontrolled proliferation. Accordingly, inactivating alterations of components of the NOTCH signaling pathway can be selected for in squamous cell carcinomas. This context-dependent dual role of NOTCH as oncogene or tumor suppressor is well documented (Nowell and Radtke, 2017).

This second level answers the question:

*In which direction does the same node act in a given context?*

It thus differs fundamentally from weight contingency. Tissue-specific tumor predisposition alone does not yet permit a decision between the two mechanisms. From the observation it follows initially neither that only the effect strength differs, nor that the sign actually changes. For sign contingency, a stronger empirical demonstration is therefore required: the same molecular state, or the same change in activity, must have opposite consequences for the malignant phenotype in different biological contexts.

#### 11.4.3 Level 3 - category contingency

The third proposed level no longer concerns merely the strength or direction of a function, but the functional identity of the node itself. In category contingency, it is assumed that the same molecular object takes on a qualitatively different function depending on its localization, its molecular state, or the cellular context, and must thereby be assigned to a different EMMI variable. A biological basis for such a possibility is provided by so-called moonlighting proteins (see the chapter 8.1). Here the same gene product can exercise several, in part biochemically largely independent, functions, without different genes or necessarily different protein isoforms being required for this (Jeffery, 1999; Jeffery, 2019; Section 8.1).

A particularly illustrative example is cytochrome c. Within the mitochondrial respiratory chain, cytochrome c functions primarily as an electron carrier between complex III and complex IV and is thus, in the proposed model, to be assigned above all to the category Energy. After release from the mitochondrion into the cytosol, the same molecule by contrast takes on a qualitatively different function: it binds to APAF1, enables the formation of the apoptosome, and thereby contributes to the activation of the caspase cascade (Liu et al., 1996). In this context it would be assigned primarily to the category Information.

This third contingency level thus answers the question:

*Which function does the same molecular node exercise at all in a given context?*

Precisely this level, however, requires a particularly restrictive application. Otherwise there would be a danger that molecular nodes are assigned to arbitrarily different categories depending on the desired explanatory goal, and that the model thereby loses falsifiability. Therefore, the following methodological rule is introduced for the model proposed here:

The primary category identity of a node is assumed to be stable as a matter of principle. A change of category assignment is admitted only when an experimentally documented qualitative change of its molecular function is present.

A category contingency should thus not be inferred merely from the fact that the significance of a protein differs between two tissues. What is required, rather, is the demonstration of an actual functional switch, such as can be observed, for example, in moonlighting proteins.

Relationship of the three contingency levels.

*Weight contingency:* The function and its direction are preserved, but their significance changes.

*Sign contingency:* The function and weighting/significance is preserved, but its effect on the phenotype under consideration is reversed.

*Category contingency:* The molecular node takes on a qualitatively different function and thereby changes its primary category assignment.

The proposed tripartition is therefore not to be understood as the description of an already-established biological law, but as a heuristic and, in principle, falsifiable systematics with which different forms of biological context-dependence can be separated from one another. Its scientific value depends essentially on the fact that different empirical criteria of demonstration apply to each contingency level, and that an assignment is not made solely on the basis of the desired explanation.

## 12 Integrative pluralism: an epistemological framework for cancer research

### 12.1 Fundamentals of integrative pluralism according to mitchell

Integrative pluralism is a philosophy-of-science approach that Sandra D. Mitchell has developed in a series of fundamental publications (Mitchell, 2002; 2003; 2009; 2023). It responds to a fundamental epistemological challenge of modern biology: The increasing complexity of biological systems and the multitude of explanatory levels necessary for their understanding can be managed neither by reductionist approaches - which seek to trace all explanations back to the molecular level - nor by simple pluralisms that allow explanatory levels to exist side by side without any connection (Mitchell, 2003).

Mitchell distinguishes three fundamental types of complexity in biological systems that are directly relevant to cancer research: (1) Dynamic complexity - state changes through nonlinear, chaotic functions; (2) Compositional complexity - the relationships between the multiple parts that constitute a more complex system; and (3) Contextual-contingent complexity - the dependence of system behavior on its evolutionary and historical development (Mitchell, 2023).

Integrative pluralism postulates that complex biological phenomena cannot be fully captured by a single explanatory level or method, but that complementary, perspectival models at different levels of organization and abstraction are both necessary and legitimate. Furthermore, these models are not interchangeable at will, but are specific to the respective research context and explanatory goal. Mitchell writes:

*“To be useful, scientific representations must leave out some features of nature to provide explanations and predictions. The flip side of partiality is that by leaving some features out, every method, model, and representation ‘selects’ features to be included. This ‘selection’ reflects a perspective - either explicitly, by identifying the features relevant to the goal of investigation, or implicitly, by the constraints imposed by the form of the representation.”* (Mitchell, 2023)

From our perspective, the four EMMI variables can be understood as an expression of the partiality described by Mitchell (2023). On the one hand, each variable represents a deliberate simplification: It summarizes a multitude of superordinate factors influencing the cell system, or the emergent property under consideration, into a manageable dimension, necessarily leaving out detailed features in the process. On the other hand, the four variables are chosen in such a way that they collectively cover the essential factors influencing the cell system - energy, matter/milieu, mechanics/structure, and information. Thus, the EMMI variables realize precisely that interplay of exclusion and inclusion that Mitchell describes as the core of partiality: It is only through the reduction to four dimensions that the model becomes explanatory and applicable, and it is precisely this reduction that simultaneously determines which aspects of the cell system are represented. In this respect, the EMMI variables are not only partial, but - in Mitchell’s sense - also perspectival: Using the four dimensions, the model selects what is to be considered essential for the description of cell transformation in the case of cancer initiation.

### 12.2 Three dimensions of multi-level research in cancer biology

O’Malley and colleagues systematically analyzed multi-level research strategies in the modern life sciences in an influential paper (O’Malley et al., 2014). They identify three dimensions that are characteristic of biological multi-level systems:

Conceptual dimension: The analyzed systems extend across multiple biological scales and require different units of analysis. In cancer research, these concern molecules, cells, cell populations, tissues, and the entire tumor ecosystem. None of these levels is privileged or fundamental in the sense that it would completely explain all other levels (O’Malley et al., 2014).

Methodological dimension: This conceptual heterogeneity requires approaches that are capable of capturing the multi-level composition and dynamics. In modern cancer research, this means the integration of genomic, transcriptomic, proteomic, metabolomic, and imaging data - a challenge that systems biology is addressing (O’Malley et al., 2014; Sauer et al., 2007).

Explanatory dimension: In multi-level systems, various activities and entities are causally linked to one another, which requires explanatory models that capture these interactions. The integration of mechanistic and mathematical models is crucial for a sound understanding of the dynamic aspects of biological systems (O’Malley et al., 2014; Mitchell, 2003; Mitchell, 2009).

### 12.3 Pragmatic laws: why biological causal relationships are not universal laws

A central argument of integrative pluralism is Mitchell’s concept of pragmatic laws. In contrast to strict physical laws - which apply universally, without exception, and by necessity of nature - biological regularities are contingent, i.e., they depend on historical and contextual conditions that have formed over the course of evolution (Mitchell, 2000; Mitchell, 2023).

Mitchell proposes not to measure biological regularities against the standard of universal physical laws, but rather by their ability to facilitate predictions, explanations, and interventions. These pragmatic laws do not apply without exception, but they do apply in defined contextual conditions with sufficient stability to be scientifically useful. Mendel’s law of segregation, for example, is contingent upon the existence of sexually reproducing organisms and an intact meiosis mechanism - conditions that emerged on Earth about two billion years ago (Mitchell, 2023).

For cancer research, this concept has far-reaching consequences: from this perspective, the somatic mutation theory does not represent a universal law of carcinogenesis, but rather a contextually limited generalization that holds true in many, but not all, cases. The growing evidence for non-mutation-based mechanisms of carcinogenesis - such as epigenetic reprogramming, chronic inflammation, the Warburg effect, and microenvironment-induced malignant transformation - is not surprising from the perspective of integrative pluralism, but rather an expression of the pragmatic nature of biological causality.

### 12.4 Integrative pluralism and the EMMI model

The EMMI model is a practical tool for implementing integrative pluralism in cancer research. It embodies three core characteristics of integrative pluralism:

1. Partiality of representation: Each EMMI variable offers a partial, perspectival view of the considered (cell) system. No single perspective is complete; only the integration of all four perspectives provides a more adequate picture of the overall system.
2. Complementarity: The four EMMI dimensions are not competing, but complementary levels of explanation. A perturbation in energy metabolism cannot be fully understood without considering the simultaneous changes in milieu, mechanics, and information.
3. Integration instead of reduction: The EMMI model does not aim for reduction to a base variable. It accepts the necessity of considering all four levels and integrating them iteratively. This approach corresponds to what Mitchell calls ongoing iterative integration in the construction of a coherent network of beliefs (Mitchell, 2023).

## 13 Systemic responses to perturbations: adaptation, reparation, and collapse

In the EMMI model, the cell system’s response to perturbations is a multi-stage, self-organized process. When one EMMI variable is perturbed, this induces adaptive responses in all other variables, as all four are highly interconnected in the scale-free network. This systemic coupling gives the cell system cohesive behavior: the resulting processes show a directed course toward a new dynamic equilibrium.

This system behavior can be illustrated using the example of hypoxia: when local tissue hypoxia occurs - for instance, due to a vascular occlusion - all four EMMI variables are immediately affected: energy (ATP depletion due to failure of oxidative phosphorylation), milieu (lactate accumulation, pH drop, ionic imbalances), mechanics (cell edema, cytoskeleton remodeling, ECM changes), and information (HIF-1α activation, induction of survival genes, inflammatory signal transduction) (Semenza, 2012; Quail and Joyce, 2013). Depending on the extent and duration of the perturbation, the systemic response leads to three possible outcomes:

Regeneration: In the case of mild, reversible perturbations, the system can restore its original relationships between the EMMI variables. The self-organization processes lead back to the original system state, as long as the organism is able to restore the original context for the tissue and its cells.

Reparation (Adaptation): In the case of moderate perturbations, the self-organization processes lead to a new, stable dynamic equilibrium that differs from the initial state. In histopathology, this corresponds, for example, to the picture of fibrosis or scarring. In the case of chronic disruptions, irreversible new structures and processes can emerge, leading to metabolic or morphological variants - a mechanism that could play a central role in chronic diseases and in carcinogenesis.

Collapse: In the case of very strong or persistent disruptions, the system collapses, which appears morphologically as necrosis. Complete collapse occurs when the cumulative destabilization of the EMMI variables overwhelms the systemic compensation mechanisms.

*Note: When looking at the pathophysiology of chronically negatively affected tissues and cells, it can be difficult to determine whether they are regenerating or repairing/adapting. For example, even higher-grade dysplastic tissue can regenerate completely. However, whether this is regeneration/adaptation or reparation, i.e., replacement by healthy cells, is not yet clear* (Yoshida et al., 2020; Wistuba et al., 1997).

For the understanding of carcinogenesis, the middle path - pathological adaptation - is of particular importance. Chronically altered environmental conditions (e.g., chronic inflammation, chronic hypoxia, chronic intoxication, hyperglycemic milieu in patients with diabetes, malnutrition) lead to the cell system reaching new, stable system states that are pathological through the continuous reshaping of its EMMI relations. From this perspective, cancer cells can be understood as cells that have entered a stable but pathological attractor due to chronic system disruptions - a state that cannot be easily reversed by individual molecular interventions, but is in principle possible (Huang et al., 2009; Hanselmann and Welter, 2016; Weaver et al., 1997).

## 14 Implications for cancer research and clinical oncology

### 14.1 Limitations of the somatic mutation theory from a systemic perspective

Since the discovery of oncogenes and tumor suppressor genes, modern cancer research has assumed that gene mutations are necessary for the development of cancer (Weinberg, 2014; Hanahan and Weinberg, 2011). Although this theory explains essential aspects of carcinogenesis and has led to significant therapeutic developments, there is so far no clear evidence that it is causative for all malignant tumors (Huang et al., 2025; López-Lázaro, 2018a; Soto and Sonnenschein, 2004; 2011).

From a systemic perspective, the somatic mutation theory is an example of a scientific generalization that Mitchell would characterize as a pragmatic law with a limited scope: It applies in many, but not all, contexts; its validity depends on historical, contextual, and systemic conditions (Mitchell, 2000; 2023).

The systemic view makes it possible to integrate important clinical observations that are difficult to explain within the framework of the somatic mutation theory: (1) the reversion of cancer cells to normal behavior through alteration of the microenvironment (context); (2) the tumor-promoting effects of chronic inflammation without direct mutagenic activity; (3) the ability of epigenetic reprogramming to establish or resolve the malignant phenotype; and (4) the clonal heterogeneity within tumors, which points to non-genetic mechanisms of phenotype diversification (Huang et al., 2025; Soto and Sonnenschein, 2011; Hanselmann and Welter, 2016; 2022; Marusyk et al., 2012).

## 15 Conclusion and outlook: systemic cancer research as a future paradigm

The concepts outlined here - emergence, dynamics, nonlinearity, variability, robustness, contingency, convergence, the EMMI variables, and integrative pluralism - are still in the early stages of medical and biological research. The systematic integration of these concepts into cancer research requires a paradigm shift: from molecular to systemic oncology. This change is not to be understood as a replacement of molecular research - whose findings remain indispensable - but as an expansion of the explanatory framework.

In practical terms, the framework becomes operationalizable as follows: by choosing an emergent property relevant to the respective question as a read-out variable - for example, the quantitative change in the DNA content of a cell or a shift in its differentiation state (cf. Sections 9.1 and 11.2 as well as BOX 1) - and by defining and holding constant a set of essential parameters from each of the four EMMI variables. Each variable can then be manipulated individually while the response of the remaining three, as well as that of the chosen emergent property, is measured. In this way it is possible to determine not only how the EMMI variables are coupled to one another, but also under which conditions and to what extent the emergent property changes. With a suitable choice of the emergent model and its parameters, this yields a first indication of how the adapting emergent property contributes to the development of a disease such as cancer - an experimental logic that does not abandon molecular biology, but makes explicit, and thereby testable, the context in which molecular findings hold.

In my second publication (Origin of Cancer: What Is a (Cancer) Cell? [Part 2]), I intend to show the manifold implications that the proposed model could have for various fields and research approaches. Here are some first topics that I will address in more detail in this context: cancer evolution, phenotypic plasticity, the ground state of cells, and others. In addition, building on the present work, I intend to examine complex cellular systems from a biological and medical perspective and to explain why cells transform under the influence of the EMMI variables.

Box 1

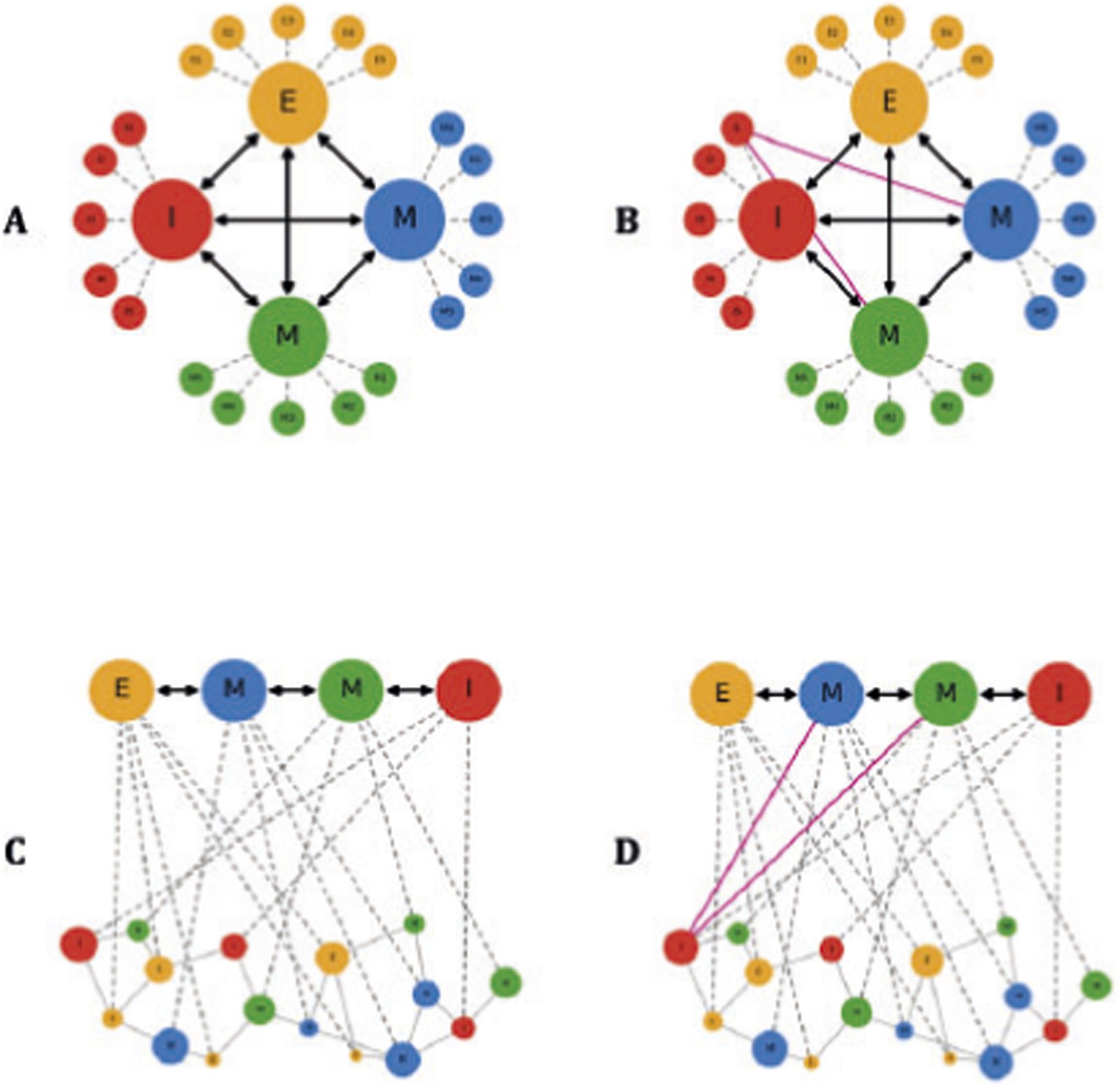

**(A,B)** Show the categorization: four categorizing nodes, to each of which nodes are assigned. Assignment relations are shown as grey dashed lines; these are not interactions. The black double-headed arrows do not represent edges of the network, but the non-orthogonality of the categories. A perturbation that begins in one category spreads to the others - and does so because the nodes brought together by the categories are connected at the level of the network. This coupling is a property of the network, not of the categorizing level.

**(C,D)** illustrate the same idea, applied to a schematically depicted measured network. The lower level represents the network itself, with color-coded nodes and edges depicted in light grey; above it lie the four categorizing nodes. Crucially, the lines between the two levels represent assignment relations and not interactions. They add no connectivity to the network; the topology of the lower part remains unaffected by the categorizing level. In **(C,D)**, the double-headed arrows are drawn only between adjacent categories: this is a linear simplification of the representation. Non-orthogonality exists between all four categories, as **(A,B)** show.

**(B,D)** differ from**(A,C)** in the pink lines. These mark individual nodes that can be assigned to more than one EMMI variable: here, I1 or I (Information) is assigned to both M (Matter/Milieu) and M (Mechanics/Structure); see also Section 3.5. This multiple assignment is not a blur in the model, but a feature of the system. Which assignment dominates is contingent - depending on tissue, developmental history, and current state (Section 9.1).

The categorization can accordingly be used in two ways. The first is didactic in nature: **(A,C)** present the model in idealized form with unambiguous assignment. The second is experimental in nature: **(B,D)** are the working version, the biologically realistic case with contingent multiple assignment. Whoever works experimentally with the model should work with **(B,D)**.

Limits of the approach: The categorization presented here is not a topological model. It makes no claim regarding the degree of connectivity, predicts no power-law exponents, and permits no quantitative network-analytic derivations. An unambiguous, context-free assignment of nodes to EMMI variables is not possible - and is not intended (Section 3.5). The value of the approach lies not in mathematical rigor, but in which questions it makes visible and which experiments it makes formulable.

## Data Availability

The original contributions presented in the study are included in the article/supplementary material, further inquiries can be directed to the corresponding author.

## In memoriam

This paper is dedicated to Loki, who never gave up.

## Glossary

Attractor: A stable state into which a dynamic system moves “on its own” and in which it remains. Cell types can be understood as such stable states; a malignant transformation corresponds to the transition into a new, pathological attractor.
Autopoiesis: The ability of a living system to continuously maintain, renew, and produce its own components. A cell itself continuously produces the components of which it is composed.
Bistability: A system can adopt two different stable states under the same conditions (e.g., “on” or “off”). Such switches explain why cells often switch between states abruptly rather than gradually.
Boolean networks: A simplified model in which each element has only two states (on/off, 1/0) and its state depends on a few other elements. It serves to understand fundamental patterns of order and self-organization in complex regulatory networks (e.g., of genes).
Categorizing hubs (EMMI categories): A term introduced in this work that is not part of established network theory. Unlike a hub, a categorizing hub is not a node of the network and has no degree of its own. It is a class over the set of nodes that groups together those nodes whose function, in a given context, is primarily determined by one of the four EMMI variables. A single node can belong to several categorizing hubs at the same time; which assignment dominates is contingent with respect to tissue, developmental history, and current state (see Box 1).
Connectivity threshold: The degree of connectivity above which a network shows a qualitatively new behavior. If the number of connections exceeds this threshold, the network spontaneously organizes itself into ordered states.
Contingency: The dependence of the current state on the individual history. What a cell is and how it reacts depends on the conditions it has previously experienced - the same stimulus can have different consequences depending on the history.
Convergence: The phenomenon that different starting points or paths lead to the same or a similar end state. In tumor biology, cells of different genetic origins can adopt similar properties in the same environment.
Downward causation: A “top-down” effect: a property of the overall system (e.g., a cell cluster) influences the behavior of its individual components (e.g., individual cells) - not just the other way around. The higher-level system thus has a downward effect on the lower-level one.
Dynamics: The property of a system to change continuously over time. Cellular processes are not fixed states, but are constantly in flux; their behavior results from the temporal interplay of many components.
Emergence: The appearance of new properties at the level of the overall system that cannot be predicted from the individual parts alone (“the whole is more than the sum of its parts”). Example: the invasive growth of a cell cluster, which no single cell possesses on its own.
Epistemological: Relating to the theory of knowledge - concerning the question of how scientific knowledge is created, justified, and validated. An “epistemological framework” therefore determines how a phenomenon can be recognized and explained in the first place.
Genuine: Real, original, inherent to the system. “Genuine complexity” refers to the actual complexity of a system that cannot be defined away.
Hubs (dominant hubs): Highly connected nodes in a network that are linked to a particularly large number of other elements. If a hub fails, the entire system is massively affected; ATP or water, for example, are such central hubs within the cell.
Hydraulic instability: An imbalance in the cell’s pressure balance (volume, internal pressure). Such physical pressure conditions can help determine cellular decisions, such as triggering programmed cell death.
Hysteresis: “History counts”: the current state of a system depends not only on the momentary conditions, but also on the path it has taken previously. Therefore, a change cannot always be reversed simply by eliminating its cause.
Nonlinearity: Cause and effect are not proportional: a small cause can have a large, unexpected effect (and vice versa). Therefore, the behavior of complex systems cannot simply be extrapolated linearly.
Non-orthogonality: The variables or categories of a model are not cleanly separable from one another, but rather overlap and influence each other (orthogonal = independent, “at right angles”; non-orthogonal = interconnected).
Oscillations: Regular, repeating fluctuations of a variable over time (rhythms). In cells, such oscillations - for example, of signaling molecules - control important processes like the cell cycle.
Partiality (of representation): A fundamental property of scientific representations (models, theories, mathematical or visual representations), according to which they always capture only a portion of their target phenomenon and necessarily omit certain features. Partiality is not a remediable incompleteness, but essential: a complete representation that included all features at all scales and all strengths of causal influences would no longer be a means of knowledge, but merely a duplicate of the phenomenon and worthless for explanation and prediction. Representations, therefore, do not map their target one-to-one but selectively capture features that are relevant or salient (especially causal) to a specific cognitive interest. Perspectivity follows directly from partiality (what is omitted simultaneously defines what is included), as does model pluralism, since different models can each represent different aspects of the same phenomenon (Mitchell, 2023).
Polyploidisation: An increase in the number of complete chromosome sets within a cell. It occurs as a programmed process during normal development (for example, in liver cells, cardiac muscle cells and the platelet-forming megakaryocytes) and permanently influences the size, metabolism and ability to divide of the cell in question. Under stress, it can also arise pathologically–in tumours in the form of polyploid giant cancer cells (PGCCs), which are significantly resistant to treatment (Darmasaputra et al., 2024; Donne et al., 2020; Derks and Bergmann, 2020).
Robustness: The ability of a system to maintain its function despite perturbations - achieved through redundancy, alternative pathways, and feedback loops. It explains, among other things, why tumors are often resistant to therapies.
Scale-free networks: Networks in which a few nodes have a large number of connections (hubs) and most nodes have only a few. Such networks are insensitive to random failures, but vulnerable to targeted attacks on the hubs.
Trajectory: The “path” of a system over time - the sequence of states it passes through one after another. In cell biology, a trajectory describes, for example, the path a cell takes through its possible states during differentiation or malignant transformation.
Variability: The natural range of fluctuation in biological systems - even genetically identical cells behave differently. This diversity is not an error, but enables adaptation; in tumors, however, it promotes the emergence of therapy-resistant cells.
